# Pay-for-Performance incentives for specialised services in England: a mixed methods evaluation

**DOI:** 10.1007/s10198-023-01630-6

**Published:** 2023-10-13

**Authors:** Yan Feng, Søren Rud Kristensen, Paula Lorgelly, Rachel Meacock, Alberto Núñez-Elvira, Marina Rodés-Sánchez, Luigi Siciliani, Matt Sutton

**Affiliations:** 1 Centre for Evaluation and Methods, Queen Mary University of London, Yvonne Carter Building, 58 Turner Street, Whitechapel, London, E1 2AB UK; 2 Institute of Global Health Innovation, Imperial College London, London, UK; 3 Danish Centre for Health Economics, University of Southern Denmark, Odense, Denmark; 4 Faculty of Medical and Health Sciences and School of Business, University of Auckland, Auckland, New Zealand; 5 Department of Applied Health Research, University College London, London, UK; 6 Health Organisation, Policy and Economics, University of Manchester, Manchester, UK; 7 Office of Health Economics, London, UK; 8 Department of Economics and Related Studies, University of York, York, UK

**Keywords:** Pay-for-Performance, Financial withholds, Programme evaluation, Mixed methods, Specialised care, English National Health Service, I12, I18, J33

## Abstract

**Background:**

A Pay-for-Performance (P4P) programme, known as Prescribed Specialised Services Commissioning for Quality and Innovation (PSS CQUIN), was introduced for specialised services in the English NHS in 2013/2014. These services treat patients with rare and complex conditions. We evaluate the implementation of PSS CQUIN contracts between 2016/2017 and 2018/2019.

**Methods:**

We used a mixed methods evaluative approach. In the quantitative analysis, we used a difference-in-differences design to evaluate the effectiveness of ten PSS CQUIN schemes across a range of targeted outcomes. Potential selection bias was addressed using propensity score matching. We also estimated impacts on costs by scheme and financial year. In the qualitative analysis, we conducted semi-structured interviews and focus group discussions to gain insights into the complexities of contract design and programme implementation. Qualitative data analysis was based on the constant comparative method, inductively generating themes.

**Results:**

The ten PSS CQUIN schemes had limited impact on the targeted outcomes. A statistically significant improvement was found for only one scheme: in the clinical area of trauma, the incentive scheme increased the probability of being discharged from Adult Critical Care within four hours of being clinically ready by 7%. The limited impact may be due to the size of the incentive payments, the complexity of the schemes’ design, and issues around ownership, contracting and flexibility.

**Conclusion:**

The PSS CQUIN schemes had little or no impact on quality improvements in specialised services. Future P4P programmes in healthcare could benefit from lessons learnt from this study on incentive design and programme implementation.

**Supplementary Information:**

The online version contains supplementary material available at 10.1007/s10198-023-01630-6.

## Introduction

Pay-for-Performance (P4P) incentive designs are widely applied in healthcare systems globally [1, 2]. These incentive schemes explicitly link provider payments to pre-defined targets such as improvements in quality of care, health outcomes or efficiency savings. P4P has been applied across different sectors of health systems [3–5]. Previous literature suggests that the effectiveness of P4P in a hospital setting is at best modest, finding either no or very small effects regardless of design factors, context or setting [3]. This limited effectiveness could be the result of variation in incentive design, implementation factors, the context in which the incentive was introduced or the methods of evaluation [6]. Despite the growing evidence with respect to the effectiveness of hospital P4P programmes, there is little research on what factors contributed to or restrained the effectiveness of a programme and how these interacted to impact effectiveness [7].

In 2013, NHS England took on the responsibility for commissioning specialised services [8]. Specialised services support people with rare and complex conditions such as rare cancers and genetic disorders [9]. Due to their specialised nature, these services are provided in relatively few hospitals by specialist clinical teams [10]. A national P4P programme called Prescribed Specialised Services Commissioning for Quality and Innovation (PSS CQUIN) was launched with the aim of improving the quality of specialised services and achieving value for money [11]. This is one of the first P4P programmes which aimed to incentivise specialised hospital services. The PSS CQUIN programme is commissioned nationally by NHS England, but managed locally by regional commissioning hubs. Between April 2016 and March 2019, the potential incentive payments available nationally totalled £900 million. In addition to incentivising specialised services, the principal features of the programme included (1) use of financial withholds rather than bonuses, (2) central development of the incentive schemes and (3) local agreements on scheme selection, performance targets and proportion of overall incentive payment allocated to each scheme.

This study provides a mixed methods evaluation of the effectiveness of the PSS CQUIN programme between 2016/2017 and 2018/2019. The quantitative evaluation focused on the performance of NHS providers with respect to ten out of a total of 35 incentive schemes within the programme. The ten schemes were selected for their strategic importance to NHS England and data availability (to support a robust empirical evaluation). In the qualitative evaluation, we interviewed key stakeholders of the programme to explore their experience throughout the period of implementation. The study contributes to the existing literature on P4P in multiple ways. First, we are not aware of any studies that evaluated P4P in the context of specialised care—complexity of care may affect the ability to respond to incentives. Second, we are one of the few studies using a mixed methods approach to evaluate the impact of a P4P scheme and offer an in-depth understanding of how the scheme was perceived and implemented. Finally, we add to the sparse evidence on the cost of implementing P4P programmes using a cost-consequences framework. Our findings offer a range of lessons for future P4P programmes and their implementation in health care.

## Background

### The National Health Service in England

The National Health Service (NHS) in England is almost entirely financed through general taxation. Funding comes from the English Department for Health and Social Care, and is managed by NHS England. Prior to July 2022 NHS England allocated the majority of its funding to over 200 Clinical Commissioning Groups (CCGs) (the 2022 Health and Care Act abolished CCGs, formalising Integrated Care Systems). CCGs (now Integrated Care Boards) were responsible for identifying the health care needs of their local populations and purchasing health care services on behalf of their population from providers such as hospitals and community healthcare service bodies. NHS England continues to directly commission some healthcare services, like specialised services.

### Specialised services

In 2012, local CCGs became responsible for commissioning emergency, elective and community care. Responsibility for commissioning specialised services was with the national body, NHS England [10]. In April 2013, NHS England became the commissioner for 143 specialised services. Specialised services are provided by teams working predominantly in teaching hospitals, large and specialist providers, to support people with rare and complex conditions [8]. There are four factors that determine whether NHS England commissions services as ‘specialised’: the individuals who require the service; its cost; staff ability to provide the service and financial implications for local purchasers [12]. The budget for specialised services in England was £14.6 billion in 2015/2016 [8].

### PSS CQUIN programme

The principle behind the PSS CQUIN design is to link a proportion of provider’s income for specialised services to their achievement of quality improvement and innovation goals. The P4P programme covers specific clinical areas known as National Programmes of Care. Each National Programme of Care has a number of incentive schemes (the number and nature of which can change over time). The schemes vary in terms of what is incentivised and the incentive design. Each year, old schemes are retired, new schemes are introduced and existing schemes are revised.

In 2016/2017, the programme included ten National Programmes of Care that offered a total of 26 PSS CQUIN incentive schemes. The majority of the schemes (19 out of 26) were introduced with the aim of improving care processes, such as targeting a reduction in cardiac surgery non-elective inpatient waiting times [11]. Some schemes incentivised structure, such as establishing and operating regional spinal surgery networks, data flows, and multiple disciplinary teams for spinal surgery patients; while others incentivised outcomes, for example involving families and carers of children and adolescents using mental health services in their care. Some schemes incentivised a combination of process and outcome or structure measures. There were 24 incentive schemes in April 2017; these included continuing, new, expanded and merged schemes, while some earlier schemes were retired [13]. These 24 schemes functioned for two years.

In total, 2.5% of the contract value for specialised services for each provider is linked to PSS CQUIN incentive metrics. The programme operates using withholds rather than bonuses, meaning that part of the contract value is withheld from a provider if they fail to meet the incentive targets.

The incentive payment is purposefully set at a level above the estimated cost to providers of delivering the incentivised care in order to ensure that incentives are high enough to induce the desired effort. In 2016/2017, the PSS CQUIN payments were developed to cover typical provider costs plus an additional 25% incentive income. For 2017/2018 and 2018/2019, payments were increased to 50% above typical provider costs [13].

PSS CQUIN incentive payments for each scheme and the list of accompanying quality metrics were centrally developed by NHS England. Providers and regional NHS England commissioning hubs then agreed locally which schemes were adopted from the national menu, the level of performance required to meet the target, and the proportion of overall PSS CQUIN payment attached to each scheme. As the national menu for the PSS CQUIN schemes is dynamic, the contracts were reviewed and agreed each time there were revisions to the national menu. The relationship between performance and payments varied from structural payments for uptake of schemes to payments directly linked to performance on specific indicators. This resulted in a complex programme of incentives.

## Methods

We applied a mixed methods approach to evaluate the PSS CQUIN programme between 2016/2017 and 2018/2019. The quantitative analysis estimated the effectiveness of ten selected schemes on their targeted outcomes as well as the impact on commissioner’s costs. The qualitative evaluation interviewed key stakeholders of the PSS CQUIN programme. The aim was to understand their perceptions of the programme, and challenges and enablers for the implementation of specific schemes.

### Quantitative evaluation

#### Criteria for scheme selection

Scheme uptake by providers and data availability for evaluation vary between individual schemes. We, therefore, did not evaluate all schemes that were implemented in 2016/2017 and 2017/2018, but instead focused on those amenable to a robust evaluation using quasi-experimental methods. This required sufficiently sized treatment and control groups, and the availability of data relating to incentivised outcomes. Focusing on those schemes meeting these data requirements ensured that our evaluation does not inappropriately attribute changes in an incentivised outcome to other factors. Our inclusion criteria for schemes to be selected included:

- There were at least ten eligible providers participating in the scheme, and at least ten that did not. This ensures that the sample size provides enough statistical power to assess the impact on the outcomes evaluated.
- Data for key variables are available for at least 1 year before and 1 year after the scheme implementation. This applies to providers in treatment and control groups.
- At least one of the scheme’s incentivised outcomes must be quantifiable in the data sets available.

We identified ten schemes that met the selection criteria for this evaluation. This includes two schemes for General services (GE1, GE2), two for Cancer services (CA1, CA3), two for Trauma services (TR1, TR3), two for Mental Health services (MH2, MH4), one for Women and Children care (WC5) and one for Internal Medicine (IM1). Eight of these schemes were implemented in April 2016, and the other two in April 2017 (CA3, WC5). Scheme MH4 was implemented in 2016, with new outcomes introduced in 2017. Following our inclusion criteria, we included MH4 in the evaluation but focused on the incentivised outcomes that were introduced in 2017.

Table 1 provides a brief description of the aims of the schemes; the 24 outcomes evaluated across the schemes are summarised in Table 2. Most of the incentivised outcomes (*n* = 17) aimed to improve efficiency by reducing length of stay (*n* = 8), reducing the volume of admissions (*n* = 8), and having fewer delayed discharges (*n* = 1). Five incentivised outcomes aim to improve clinical quality by reducing waiting times, readmission rates, safety events during admission, night discharges, and cancellation for non-elective surgeries. Two incentivised outcomes aimed to reduce mortality within 30 days of hospital treatment.

**Table 1:** Implementation periods and brief descriptions of the aims of the ten PSS CQUIN schemes evaluated in this study

National programmes of care	Schemes	2016/2017	2017/2018 and 2018/2019	Brief descriptions of aims
General Schemes	GE1	x	x	Implement clinical utilisation review for reduction in inappropriate hospital utilisation
	GE2	x	x	Use of the patient activation measure survey to improve outcomes
Cancer	CA1	x		Improve access for patients with incurable cancer to enhanced supportive care
	CA1/IM1		x	Improve access for patients with incurable cancer/hepato-pancreato-biliary to enhanced supportive care
	CA3		x	Optimise decision-making for patients with palliative treatment
Internal Medicine	IM1	x		Reduce waiting times for patients referred for coronary artery bypass grafting
Trauma	TR1	x		Reduce delayed discharges from adult critical care
	TR3	x	x	Establish multi-disciplinary teams (MDTs) to sanction referrals for surgery, with data entering
Women and Children	WC5		x	Optimise the use of neonatal care through improve community support
Mental Health	MH2	x	x	Deliver education and training courses to complement treatment
	MH4		x	Remove hold-ups in discharge

^a^Table was reproduced from Table 1 in Feng et al. (2019) [11]

**Table 2:** Number of eligible providers, incentivised outcomes evaluated, financial incentives, and data source for evaluation by scheme

National programmes of care	Schemes	No. of eligible providers (N took up the scheme)	Incentivised outcomes evaluated	Key financial incentives elements	Data source
General Schemes	GE1	59 (43)	1. LOS for emergency admissions 2. LOS for elective admissions 3. N of emergency admissions 4. N of unique patients treated	The general principle to award providers is to pay their achievements on each incentivised activity	Hospital Episodes Statistics (HES) Admitted Patient Care (APC) and Clinical Utilisation Review (CUR) data from NHS England
	GE2	164 (35)	1. N of HIV emergency admissions 2. N of respiratory disease emergency admissions 3. N of renal disease emergency admissions 4. Emergency readmissions within 30 days (respiratory patients)	2016/2017: £50,000/provider (> = 500 patients) 2017/2019: For each provider, one payment calculation method applied to existing patient groups and another method applied to new patient groups	HES Outpatient Care (OPC)
Cancer	CA1	144 (22)	1. N of chemotherapy or radiotherapy treatments 2. N of emergency admissions 3. LOS of emergency admissions	2016/2017: £500/patient (< 800 patients) 2017/2019: £600/patient	HES APC and HES Adult Critical Care (ACC)
	CA3	142 (33)	1. N of deaths within 30 days of the last chemotherapy for any patient that received specialised cancer service	£35,000 + £40/patient	HES APC
Internal Medicine	IM1	28 (12)	1.Waiting time within 7 days 2. Total LOS (Coronary Artery Bypass Graft) 3. Mortality within 30 days 4. Patient safety events during the admission	£10,000 + £150/reduced waiting day	HES APC
Trauma	TR1	135 (63)	1. Delayed discharges < 4 h 2. Night discharges 3. Urgent operations cancelled	Two payment approaches applied, with one approach as default and the other one applied to selected trusts	HES ACC and Urgent Operations Cancelled from NHS England
	TR3	42 (14)	1. N of spinal surgery patients treated	2016/2017: £50,000/network + £150/patient 2017/2019: £60,000/network + £180/patient	HES APC
Women and Children	WC5	41 (19)	1. Hospital LOS for infants born prematurely	£200,000/Outreach Team	HES APC
Mental Health	MH2	72 (32)	1. LOS between 90 and 365 days	2016/2017: £10,000/provider + £2000/patient 2017/2019: £12,000/provider + £2,400/patient	North of England Commissioning Support Unit
	MH4	103 (40)	1. LOS for children and adolescents 2. LOS for adults	2017/2019: Adjusted X% contract value + CUR costs	North of England Commissioning Support Unit

2017/2019 in the 5th column refers to financial years 2017/2018 and 2018/2019

The 5th column was reproduced from Table 1 in Feng et al. (2019) [11]

*LOS* length of stay, *N* number

#### Data

Much of the analysis for the effectiveness evaluation relied on Hospital Episodes Statistics (HES), an administrative data set capturing all hospital activity in England. We used the admitted patient care (APC), outpatient care (OPC), and adult critical care (ACC) data sets. In addition, we made use of the Mental Health data from the North of England Commissioning Support Unit, Urgent Operations Cancelled data from NHS England, and Clinical Utilisation Review (CUR) data from NHS England. NHS England also provided data for the cost-consequences analyses.

#### Methods for effectiveness evaluation

We use an event study difference-in-differences design to assess the impact of each PSS CQUIN scheme on the incentivised outcomes. We compare the change in outcomes before and after the scheme was implemented among providers taking up a specific scheme (treatment group) with eligible providers not taking up the scheme (control group). We estimate the following model:

1 $${y}_{iht}=\alpha + {{\varvec{d}}}_{t}+({T}_{h}*{{\varvec{d}}}_{t}){{\varvec{\beta}}}_{t}+{{\varvec{X}}}_{iht}{\varvec{\delta}}+{{\varvec{d}}}_{h}+{\epsilon }_{iht,}$$

where ${y}_{iht}$ is the incentivised outcome for patient *i* who was treated by provider *h* in year *t*. ${{\varvec{d}}}_{t}$ is a vector of year dummies to allow for a time trend (e.g. due to improvements in incentivised outcome over time as the results of new technologies and other factors). ${T}_{h}$ is a dummy variable equal to one if provider *h* took up a particular PSS CQUIN scheme of interest (incentivised providers in treatment group), and zero otherwise (non-incentivised providers in the control group). ${{\varvec{X}}}_{iht}$ is a vector of control variables at the patient level that includes age, gender, gender interacted with age, and Charlson comorbidities index. ${{\varvec{d}}}_{h}$ is a vector of provider fixed effects, which account for time-invariant unobserved factors at the provider level. ${\epsilon }_{iht}$ is the error term.

In Eq. (1), we allow for the outcomes in the treatment group to vary in each year which enables testing of the parallel trends assumption as well as the estimation of impact. We use the year before the scheme was implemented as the reference year. The reference year is 2015/2016 for most schemes except for schemes CA3, WC5 and MH4, where it is 2016/2017. For most outcomes we evaluated, the pre-policy period started from 2012/2013 except for one outcome under TR1 and two outcomes under MH4 due to data availability. Difference-in-differences methods rely on the parallel trends assumption. The null hypothesis is that the vector of coefficients ${{\varvec{\beta}}}_{t}$ for the pre-implementation period (jointly or per year) equal to zero (so that the parallel trends assumption holds). In contrast, we expect the vector of coefficients ${{\varvec{\beta}}}_{t}$ for the post-implementation period to be statistically significant if the scheme had an effect on performance. For schemes that were retired in 2017/2018, we still included 2017/2018 as a post-implementation year to test whether the schemes had any sustained effect.

The dependent variable in Eq. (1) is measured at the *patient* level, and this is the approach for the majority of outcomes.

For some schemes the outcomes are only available at the *hospital/Trust* level, for example number of emergency admission under General Schemes 1 (GE1) was reported at Trust level. In these instances, we estimate a modified Eq. (1):

2 $${y}_{ht}=\alpha + {{\varvec{d}}}_{t}+\left({T}_{h}*{{\varvec{d}}}_{t}\right){{\varvec{\beta}}}_{t}+{{\varvec{X}}}_{ht}{\varvec{\delta}}+{{\varvec{d}}}_{h}+{\epsilon }_{ht},$$

where patient covariates are averaged at the provider level. In the results section we indicate whether the analysis was conducted at patient or provider level.

NHS England commissioners negotiate annual contracts with providers, and offer a tailored PSS CQUIN package to each provider as part of their contract. The package does not necessarily include all schemes that the provider is eligible for. A relevant scheme will not be offered if any of the following hold: (1) the incentivised behaviour is business-as-usual for the provider; (2) the expected costs exceed the expected benefits to patients and commissioner or (3) PSS CQUIN funds are exhausted on other schemes which are assumed to deliver better value from this provider.

While it is voluntary for a provider to accept or reject the PSS CQUIN package offered, providers felt they had limited scope to refuse commissioners’ offers (see below Sect. “Qualitative evaluation”). By rejecting the package, providers would forfeit an opportunity to raise revenues, though this may be an efficient decision if they expect the cost of implementing the scheme to exceed the additional revenues. It should be noted that providers could accept the contract package but then exert no effort on schemes that they felt unable to achieve. This feature and the fact that the selection of schemes from a national menu is negotiated locally could potentially introduce selection bias. We apply a propensity score matching approach to minimise such selection bias.

Amongst all eligible providers, we regress whether a provider joins the scheme or not against a set of provider characteristics and use nearest neighbour matching without replacement to match incentivised providers to providers that did not join the scheme. Variables in the matching equation include total number of beds in the hospital, proportion of doctors out of the total number of hospital staff, the Market Forces Factor index and binary indicators for whether a provider had Foundation Trust status, was a teaching hospital, or was located in London. In the instances where we find significant differences in characteristics between the providers taking up the scheme and the wider group of control providers, we present the estimation results after matching.

Where appropriate, we present results from ordinary least squares (OLS) estimation. When the dependent variable is binary or count, an appropriate estimator such as a logistic regression or the negative binomial model was applied for the primary analysis. Standard errors are clustered by provider in the patient level models.

#### Methods for the cost-consequences framework

We estimated the impact on costs for the ten schemes from a commissioner’s perspective. A feature of the PSS CQUIN programme is that it is based on withholding a proportion of funds from providers rather than offering additional payments. The incentive payments represent costs attributable to the programme. However, depending upon the perspective taken and comparator, the withhold nature means that the difference between the level of incentives available and payments could be viewed as a cost saving compared to the counterfactual scenario in which the PSS CQUIN programme had not been implemented.

When estimating the costs to commissioners of PSS CQUIN schemes, we distinguish between three categories of costs: the incentive payments, the scheme implementation costs and the cost of central commissioner time administering the schemes. Incentive payments refer to the monetary value paid by commissioners to providers for meeting the pre-defined targets. Implementation costs were only available for schemes GE1 and GE2; these were payments made by NHS England to consultancies helping implement these schemes. The costs of central commissioner time were calculated as the salary costs for time staff dedicated to the PSS CQUIN programme. The time spent on each scheme was calculated as a proportion of the total time spent on the PSS CQUIN programme, where this proportion was proxied by the ratio of planned contract value for the scheme relative to the total planned contract value for all schemes in the year.

For any incentivised outcome where we found an improvement in outcome as the result of the PSS CQUIN scheme, we proposed to compare costs and consequences. Where possible we monetarised the consequence and presented this as efficiency savings. For example, length of stay (LOS) was valued using the cost of a bed day to estimate the value of bed days saved. Efficiency savings are then compared with commissioner’s costs.

All analyses were conducted using STATA/MP 16.0.

### Qualitative evaluation

The qualitative component of the evaluation captured the experiences of commissioners and providers with respect to the adoption and implementation of PSS CQUIN schemes. Using semi-structured interviews and focus group discussions, we aimed to explore issues with respect to the contract design, scheme uptake and barriers and enablers to implementation of the schemes. The interview schedules are presented in Supplementary Appendix 1.

Purposeful recruitment involved: (1) NHS England initially identifying local NHS commissioners who had broad experience with the PSS CQUIN schemes, (2) local commissioners in different regions nominating relevant individuals in NHS Trusts and (3) a snowballing recruitment technique, where individuals were asked to nominate others in the Trust (be it administrative or clinical) to take part in the study. National Programmes of Care and Clinical Reference Groups (CRG) members were also identified using a similar snowballing approach. Recruitment continued until data saturation was reached.

Analysis of the qualitative data was based on the constant comparative method, inductively generating thematic categories [14]. We analysed commissioner data separately from provider data to ensure that each were understood in terms of their relevant context; we then integrated qualitative data, focusing on synergies and divergent views, to gain insight on the complexities underlying the contracting/delivery processes. This process allowed us to produce a comprehensive picture of the experiences around the implementation of the schemes at different levels. Audio-recordings were transcribed verbatim and then reviewed against the transcriptions. Field notes of group and individual interviews were also reviewed. As data were collected, thematic analysis was undertaken in an iterative process. The validity and reliability of the theme development was confirmed using a second coder, who was also present during the interviews and focus group discussions. Coding was discussed to reach consensus around the final key themes. To assist with thematic analysis, data were coded with the QDA Miner Lite v2.0 software.

After obtaining sponsorship and insurance from the Joint Research Compliance Office at Imperial College London, ethical and research governance approval for this project were obtained from the Health Research Authority (IRAS Project ID: 244930).

## Results

### Effectiveness evaluation

**Fig. 1:**
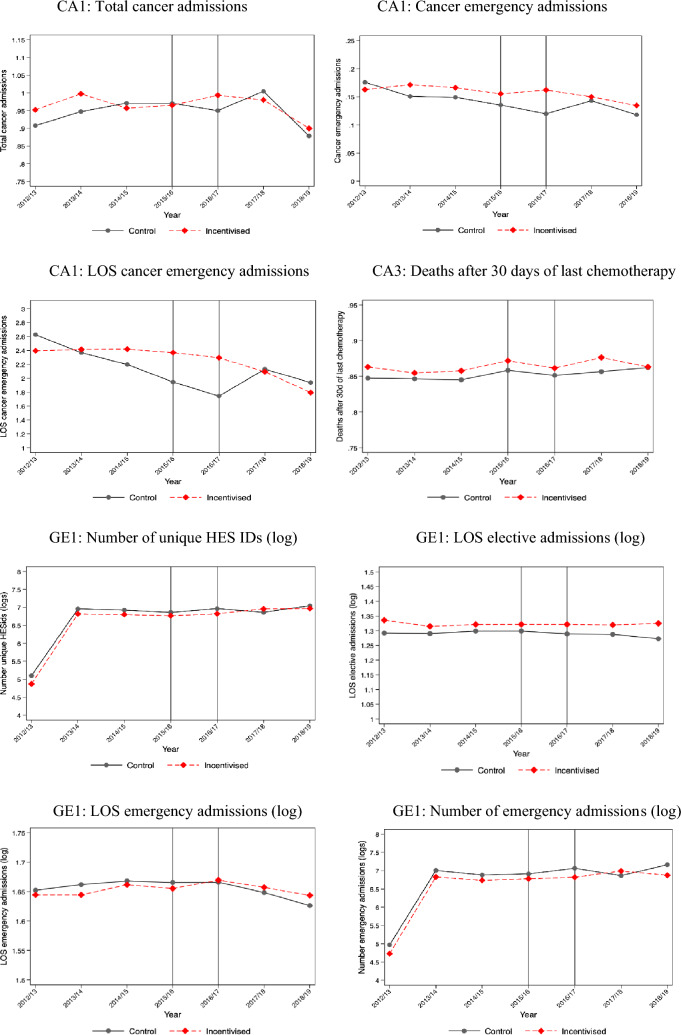

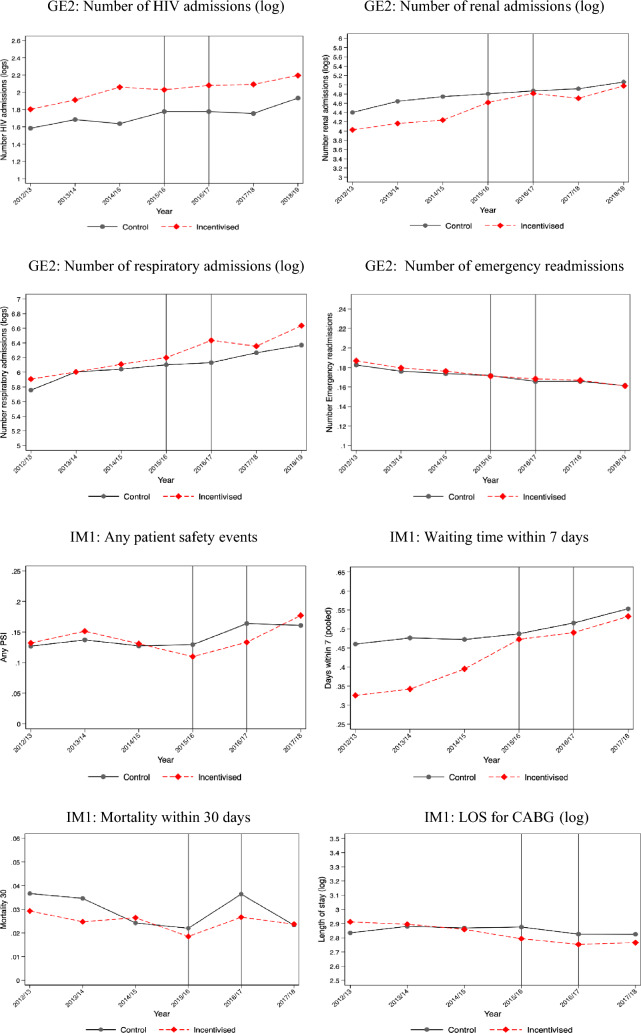

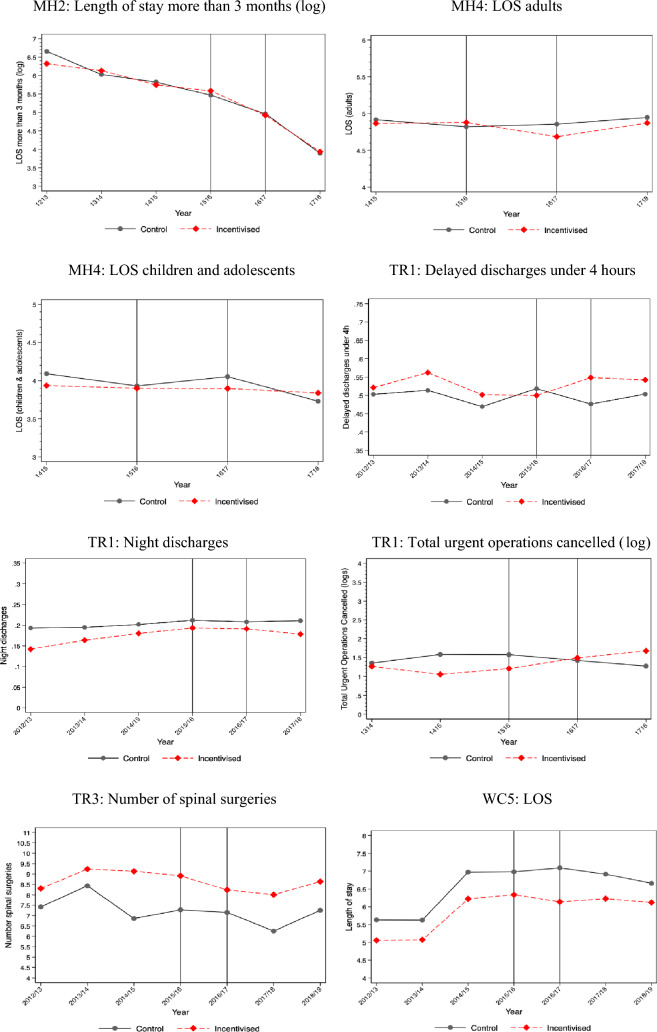
Visual summary of the 24 incentivised outcomes for providers in treatment and control groups over time

**Fig. 2:**
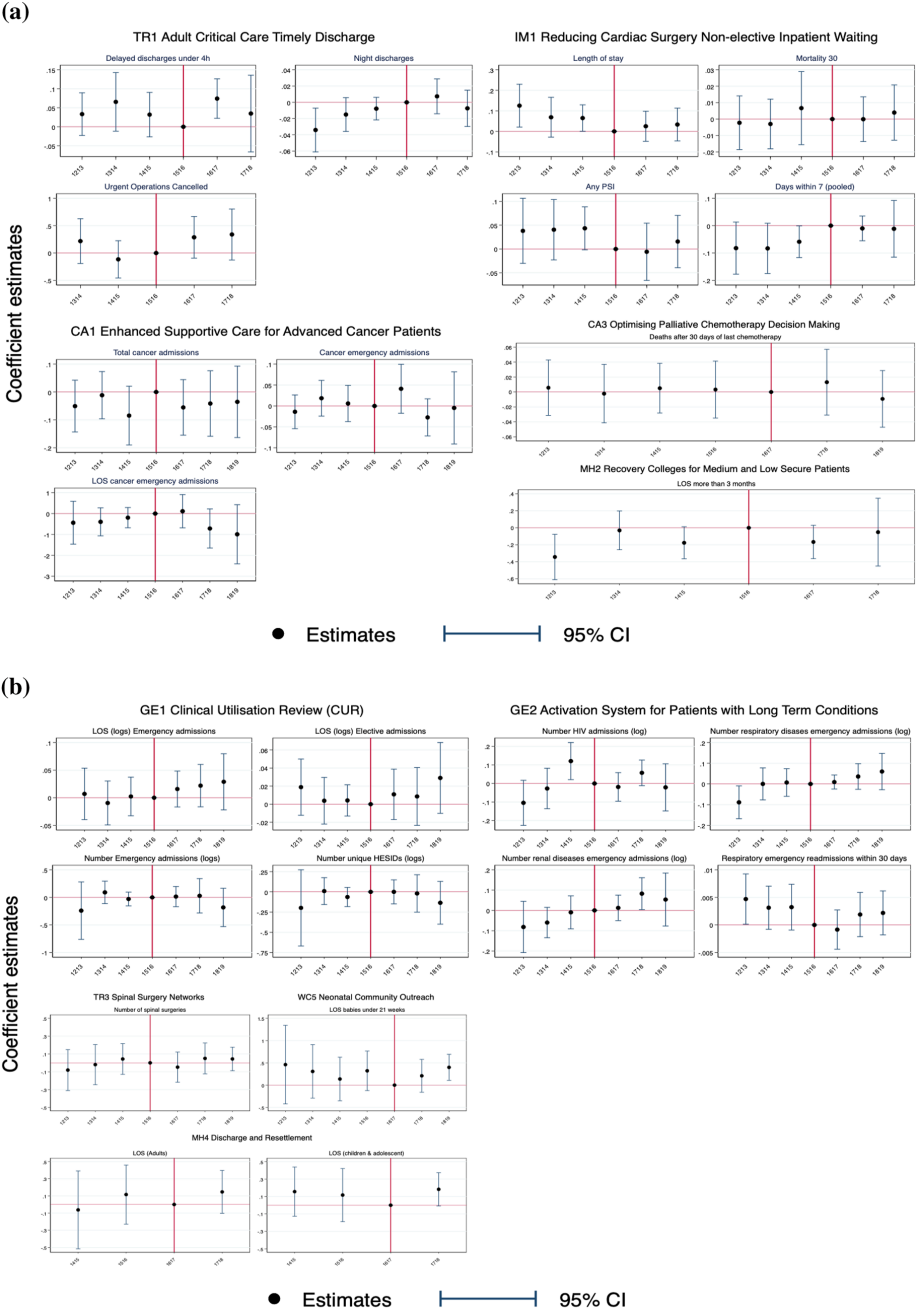
Visual summary of the estimated coefficients from the interaction terms of year dummies and a dummy for providers being in the treatment versus the control group. The red vertical lines indicate the reference year (2015/2016 for most instances). There are 24 figures in total with one figure for each evaluated outcome

The descriptive statistics of treatment and control providers by scheme are presented in Supplementary Appendix 2. The providers in the treatment and control groups are different for six of ten schemes, but these differences diminish substantially after matching. For two schemes (GE1 and GE2), the provider groups were similar without matching. We do not have provider characteristics data for two schemes (MH2 and MH4) because the information is not available from NHS England. Figure 1 shows changes in each incentivised outcome for providers in treatment and control groups over time. We report the mean value for each incentivised outcome before and after the introduction of PSS CQUIN schemes in Supplementary Appendix 3. Table 3 presents the results of the effectiveness evaluation of ten PSS CQUIN schemes. In columns 3–5, we report the coefficients on the interaction of a dummy for providers being in the treatment group and the respective year. The 6th column presents the statistical significance of the joint test of pre-intervention year dummies for parallel trends assumption. The 7th column identifies whether our estimation was based on a matched sample. The final column indicates whether the data used for each evaluation was at NHS Trust or patient level. Figure 2 provides a visual summary of the evaluation results from the 10 schemes and 24 outcomes.

**Table 3:** Results from evaluating the effectiveness of ten PSS CQUIN schemes

	*N*	2016/2017	2017/2018	2018/2019	Joint sig of pre-trend	Matched sample	Trust level analysis
		Coefficients (std error)	Coefficients (std error)	Coefficients (std error)	*F* test of joint significance		
GE1: clinical utilisation review							
Log of LOS emergency admissions	3,483,683	0.016 (0.016)	0.022 (0.018)	0.029 (0.024)	0.069	No	No
Log of LOS elective admissions	1,305,924	0.011 (0.013)	0.009 (0.015)	0.029 (0.019)	0.562	No	No
Log of number of emergency admissions	1901	0.014 (0.088)	0.029 (0.150)	− 0.182 (0.167)	0.115	No	Yes
Log of number of unique patients treated	1897	0.000 (0.071)	− 0.019 (0.111)	− 0.135 (0.127)	0.382	No	Yes
GE2: activation system for patients with long-term conditions							
Log of number of HIV emergency admissions	10,238	− 0.019 (0.039)	0.057 (0.035)	− 0.021 (0.064)	0.016*	No	No
Log of number of respiratory disease emergency admissions	12,802	0.009 (0.017)	0.036 (0.031)	0.060 (0.044)	0.097	No	No
Log of number of renal disease emergency admissions	10,092	0.012 (0.032)	0.082* (0.040)	0.053 (0.066)	0.328	No	No
Emergency readmissions within 30 days (respiratory patients)	9,634,377	− 0.009 (0.020)	0.021 (0.022)	0.024 (0.022)	0.204	No	No
CA1: enhanced supportive care for advanced cancer patients							
Number of chemotherapy/radiotherapy treatments	39,495	− 0.055 (0.051)	− 0.041 (0.060)	− 0.035 (0.065)	0.487	Yes	No
Number of emergency admissions	39,495	0.041 (0.030)	− 0.027 (0.023)	− 0.005 (0.044)	0.339	Yes	No
LOS emergency admissions	39,495	0.111 (0.394)	− 0.715 (0.463)	− 0.991 (0.701)	0.689	Yes	No
CA3: optimising palliative chemotherapy decision-making							
Deaths within 30 days of last chemotherapy	18,605		0.013 (0.022)	− 0.009 (0.019)	0.992	Yes	No
IM1: reducing cardiac surgery non-elective inpatient waiting							
Days within 7 (pooled)	14,355	− 0.010 (0.023)	− 0.011 (0.053)		0.220	Yes	No
Length of stay or LOS (coronary artery bypass graft)	14,355	0.025 (0.035)	0.033 (0.039)		0.035*	Yes	No
Mortality within 30 days	14,355	− 0.0001 (0.007)	0.004 (0.009)		0.771	Yes	No
Any Patient safety incidents	14,355	− 0.006 (0.031)	0.016 (0.028)		0.169	Yes	No
TR1: adult critical care timely discharge							
Delayed discharges < 4 h	708,837	0.074** (0.026)	0.035 (0.052)		0.390	Yes	No
Night discharges	708,798	0.007 (0.011)	− 0.007 (0.011)		0.071	Yes	No
Urgent operations cancelled	1714	0.287 (0.191)	0.339 (0.234)		0.178	Yes	Yes
TR3: spinal surgery networks							
Number of spinal surgeries	3424	− 0.049 (0.086)	0.050 (0.088)	0.043 (0.067)	0.487	Yes	Yes
WC5: Neonatal Community Outreach							
LOS of newborns	68,959		0.209 (0.180)	0.399** (0.143)	0.526	Yes	No
MH2: recovery colleges for patients who receive low and medium secure mental health services							
Log of LOS	7424	− 0.166 (0.098)	− 0.051 (0.199)		0.015*	No	No
MH4: discharge and resettlement planning programme in MH (to remove hold-ups in discharge)							
Log of LOS children and adolescents	5009		0.183 (0.095)		0.493	No	No
Log of LOS adults	2274		0.147 (0.124)		0.494	No	No

Estimates from OLS regression at the patient level (unless otherwise indicated). Regressions include provider fixed effects and controls for patient characteristics. Standard errors are clustered by provider in the patient level models. The reference year is the year before the scheme implementation: 2015/2016 for most of the cases except for schemes CA3, WC5 and MH4 where it is 2016/2017

Negative binomial regression applied to estimate the impacts on number of chemotherapy/radiotherapy treatments and number of emergency admission under scheme CA1

Logistic regression applied to estimate the impact on deaths within 30 days of last chemotherapy under scheme CA3

*p < 0.05 **p < 0.01 ***p < 0.001

Table 3 shows that among the 24 incentivised outcomes, only in three cases were the PSS CQUIN schemes associated with a statistically significant change in outcomes. The three outcomes came from three different PSS CQUIN schemes: TR1 in the Trauma Programme, GE2 in the General Schemes Programme, and WC5 in the Women and Children Programme. Improved outcomes were identified in just one scheme (TR1), with negative impacts detected on outcomes in the other two schemes.

We estimated that the Trauma Programme scheme TR1, which incentivised timely discharge from Adult Critical Care, increased the probability of hospital discharge from Adult Critical Care occurring within 4 h of being clinically ready by 7% (pre-policy mean is 52%) in 2016/2017. This improvement was not sustained in 2017/2018 when the scheme was retired. In addition to the primary outcome, we also evaluated the impact of the scheme on two secondary outcomes: nightly discharges and cancelled urgent operations. Table 3 shows no statistically significant effect of the incentive programme on these outcomes.

Scheme GE2 in the General Schemes Programme linked lump sum incentive payments to the following structural investments: (1) implementing the use of a patient activation measurement (PAM) survey instrument to assess patient competences in self-managing long-term conditions and (2) the rollout of activation interventions with the aim to raise patient activation levels. We evaluated two expected outcome improvements (the number of patients admitted to emergency care and hospital readmission within 30 days) for patients with HIV, respiratory, and renal disease (those targeted by the PAM survey). We only found a statistically significant effect on the volume of patients being admitted from participating Trusts in the second year of the scheme (2017/2018) for patients with renal disease; however, notably, this was an increase in emergency admissions, contrary to the scheme’s intention.

Scheme WC5 in the Women and Children Programme aimed to reduce LOS for premature newborns, through the improvement of community nursing support, with the additional aim of freeing up capacity in special care cots. The incentive scheme included a lump sum payment for each new neonatal community outreach team which is a collaboration between Neonatal Intensive Care providers, their local Operational Delivery Networks, Neonatal Intensive Care Unit and Special Care Baby Unit. We evaluated the impact of this scheme on the LOS for newborns under hospital neonatal critical care. We found a statistically significant increase in the LOS for newborns at incentivised providers in the second year of the scheme (2018/2019), again contrary to the intentions of the incentive scheme.

We did not detect statistically significant effects (at 5% level) on the remaining 21 outcomes. Among the 21 outcomes, 14 of them showed the effects were contrary to the scheme intentions. Our event-based difference-in-differences approach found that incentive payments of the type offered to NHS Trusts under the PSS CQUIN programme had no effect on:

- the number of emergency admissions, LOS of emergency and elective admissions, and the number of patients treated, when incentivised to provide clinical utilisation review (GE1);
- the number and LOS of advanced cancer patients having emergency admissions, and number of chemotherapy/radiotherapy treatments, when incentivised to provide early supportive care (CA1);
- deaths within 30 days of chemotherapy treatment, when incentivised to optimise palliative chemotherapy (CA3);
- cardiac surgery waiting times, LOS, mortality and patient safety, when incentivised to improve the care pathway for patients referred for coronary artery bypass grafting (IM1);
- the number of spinal surgeries, when incentivised to create regional networks and multi-disciplinary teams (TR3);
- the LOS of adult mental health patients who receive low and medium secure mental health services, when incentivised to provide education and training courses to complement mental health treatment (MH2);
- the LOS of young people in mental health units and adult mental health patients, when incentivised to deliver discharge and resettlement plans (MH4).

### Cost-consequences analysis

Table 4 summarises the commissioner costs by scheme and financial year for the ten PSS CQUIN schemes we evaluated. Across the three types of costs, the largest costs were those associated with the incentive payments themselves, with a total of £150.784 m of incentive payments paid out to providers between 2016/2017 and 2018/2019 Q3. A 100% compliance with all payment targets in a scheme is linked to full award of the PSS CQUIN scheme value to providers. Partial compliance awards providers with a proportional payment. In 2016/2017, providers were awarded £45.926 m from the seven PSS CQUIN schemes that we evaluated out of the total of £61.592 m plan value attached to these schemes (75%). The achievement increased to 92% in 2017/2018 (awarded £60.772 m from eight schemes with a total value of £65.797 m). Depending upon the perspective taken, the incentive payments awarded to providers could be viewed as costs of the programme. Alternatively, the difference between these payments and the plan value could be seen as cost savings associated with the schemes compared to a counterfactual situation in which the scheme was absent. The scheme with the highest plan value and incentive payments awarded was GE1 which incentivised the implementation of clinical utilisation reviews to reduce inappropriate hospital utilisation.

**Table 4:** Commissioner costs for the ten PSS CQUIN schemes (£)

National programmes of care	Schemes	Incentive payments			Implementation costs			Commissioners’ time costs			Total
		2016/2017	2017/2018	2018/2019	2016/2017	2017/2018	2018/2019	2016/2017	2017/2018	2018/2019	
General Schemes	GE1	16,567,205	18,380,575	13,555,710	210,000	210,000	280,000	9361	9923	10,482	49,233,256
	GE2	2,363,335	2,677,983	869,525	NA	102,059	244,941	1050	1345	577	6,260,815
Cancer	CA1	5,212,438	4,983,195	2,361,907	NA	NA	NA	2038	2493	1726	12,563,797
	CA3	–	3,966,479	3,679,230	–	NA	NA	–	2322	2157	7,650,188
Internal Medicine	IM1	1,588,300	–	–	NA	–	–	620	–	–	1,588,920
Trauma	TR1	10,840,621	–	–	NA	–	–	5526	–	–	10,846,147
	TR3	1,707,068	4,847,902	3,528,910	NA	NA	NA	780	2306	1959	10,088,925
Women and Children	WC5	–	5,621,773	3,764,450	–	NA	NA	–	2806	2750	9,391,779
Mental Health	MH2	7,646,724	9,392,471	8,007,474	NA	NA	NA	2768	4810	3982	25,058,229
	MH4	NA	10,901,957	8,319,010	NA	NA	NA	NA	5850	4201	19,231,018
Total paid PSS CQUIN value	All 10 schemes	45,925,691	60,772,336	44,086,216	210,000	312,059	524,941	22,142	31,856	27,834	
Total planned PSS CQUIN value	All 10 schemes	61,591,528	65,797,119	55,977,888							

For 2018/2019, incentive payments include first three quarters only as the payments made to providers in the last quarter of 2018/2019 were not available to the project team

Total planned PSS CQUIN value for incentive payments on all 10 schemes includes four quarters of 2018/19

NA means data were not available to the project team

“–” means schemes were not active

Total planned PSS CQUIN value for all schemes in 2018/2019 was not available to the project team. The value was required for calculating Commissioners’ time costs. Value in 2016/2017 was applied

As reported in Sect. “Effectiveness evaluation”, under the Trauma Programme scheme TR1, patients treated by incentivised providers experienced an improvement of 7% in the probability of being discharged from Adult Critical Care within 4 h of being declared clinically ready. Assuming this finding was not merely due to gaming, but reflects actual changes in LOS, we estimate this is an average reduction in discharge delays of 2 h per patient. Given 86,813 critical care patients were admitted to incentivised providers in 2016/2017, this amounts to a likely reduction in critical care admission time of 178,647 h. In bed days saved this equates to efficiency savings of £1.6 m, using the weighted average of the national average unit costs of adult critical care bed days in 2016/2017 (£211). Table 4 shows that for TR1 scheme, commissioners incurred costs of £5526 in staff time. If only these staff costs are considered, the efficiency savings from bed days saved far outweigh the cost of this staff time. However, from the other perspective the costs to commissioners of this scheme as a whole (£10.8 million), including incentive payments and staff time costs, outweigh this efficiency savings (£1.6 m).

We did not detect improvements for any of the other 23 outcomes, hence no other cost-consequences analyses were undertaken.

### Qualitative evaluation

**Fig. 3:**
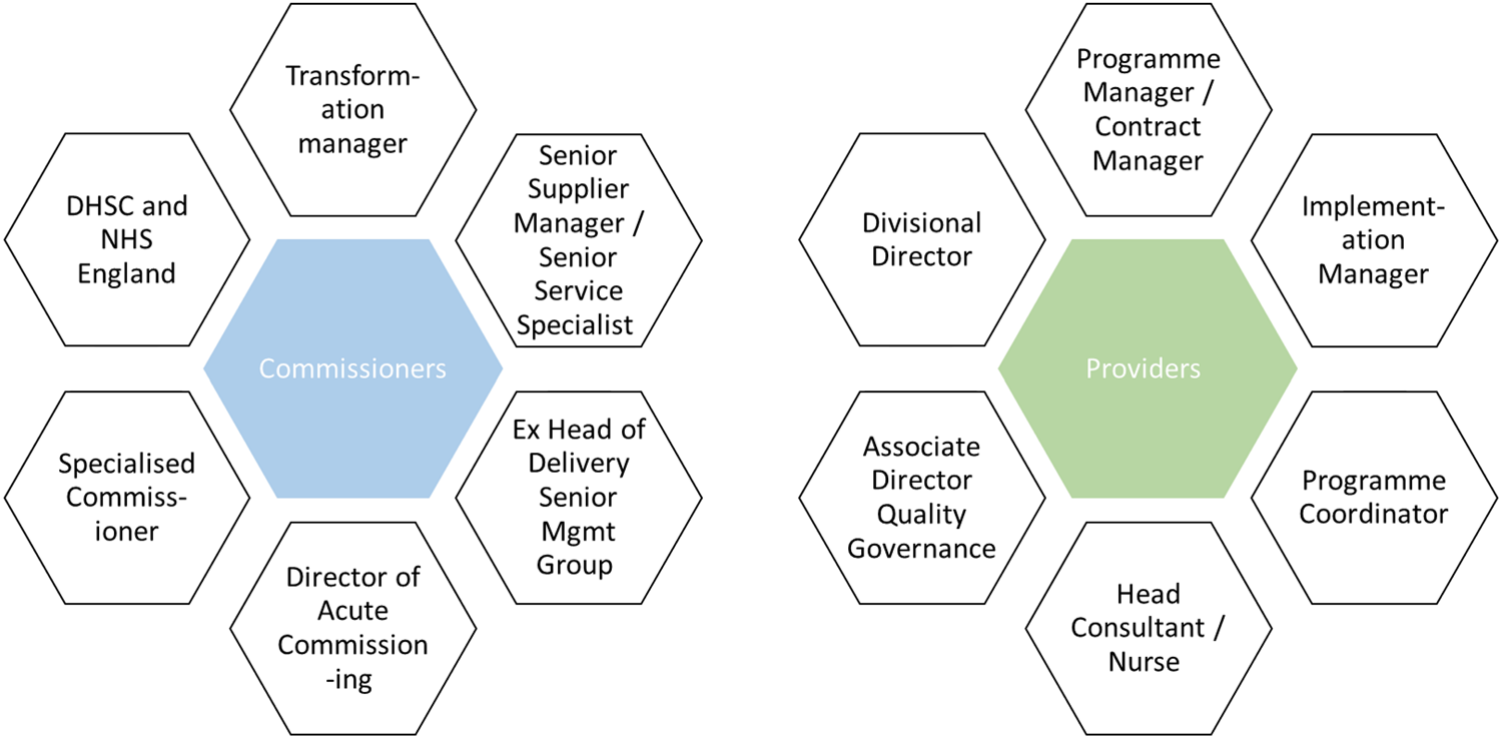
Job titles for participants in the semi-structured interviews and focus group discussions by commissioners and healthcare providers

Interviews for the qualitative evaluation were undertaken between August 2018 and December 2019. A total of 28 participants took part, with a balanced representation of commissioners and providers. In-depth semi-structured interviews were conducted with commissioners, including NHS England (*n* = 6), local commissioners (*n* = 6) and National Programmes of Care members (*n* = 2). One semi-structured interview (*n* = 1) and three focus group discussions (*n* = 13) were carried out with providers based in different clinical and administrative departments, covering four National Programmes of Care (General Medicine, Blood and Infection, Internal Medicine, and Mental Health). We purposively sampled individuals within provider organisers who had clinical patient-facing roles, given the CQUIN schemes ultimately focus on improving the patient experience. Half of the respondents were clinical. Figure 3 details the specifics of the participants.

The qualitative analysis yielded insights on participants’ perceptions of the value of PSS CQUIN schemes, and on the incentivised outcomes of the programme. Exploring uptake, implementation, benefits and any broader effects of the schemes, the following seven key themes were identified: communication; design of the schemes; ownership; timeliness and time constraints; flexibility of the schemes; credibility of incentives and sustainability. The context of each theme is described below and verbatim quotes from commissioners [C] and providers [P] offer examples of the issues discussed. Often, the issues identified are not mutually exclusive and many of the themes and issues overlap. Some of the themes that emerged could be described as barriers to effective contracting and implementation and, therefore, offer learnings with respect to the absence of evidence of effectiveness found in the quantitative analysis.

#### Communication

A lack of effective written and verbal communication between commissioners and providers (including the clarity of scheme documentation) was identified as one of the main barriers to ensuring a favourable reception by providers. Participants underscored that some schemes were too ambitious, documents lengthy, unclear or unorganised, which led to confusion during implementation. “[The documentation was] never very clear, [you] always have to read lots of detail into the actual text as to what you are meant to be doing, there is no summary at the end with a timeline … finding that we have read it in lots of detail and we’ve missed something or we have misinterpreted something and there is no one to ask questions to, or if we ask [commissioners] we don’t get answers back.” [P1].

Participants specifically highlighted difficulties in the approach to involving providers in the process, and the need to draw other stakeholders into the process: “there’s got to be really robust involvement with specialists in those areas, whether it’s public health, clinicians, people who understand what the effect you’re trying to achieve is.” [C1].

#### Design of the schemes

An implication of a lack of clear communication is that on receipt of the PSS CQUIN schemes providers sometimes struggled to understand what was required and how exactly this should be done. “Sometimes clinicians feel [the schemes] don’t measure what they are supposed to measure, and that there are quite artificial things to be answered. They are thought of by people who are outside of the service usually and then are imposed on us so it’s not really what shows we’re doing well.” [P3] The suggestion was that the lack of clarity in defining the schemes was in part due to the design of the schemes: “They [designers] will say, ‘this is the benefit and there’s an evidence base’, but then they are struggling to see exactly how we’re going to pay, what measurement we’re going to use.” [C4].

#### Ownership

There appeared to be confusion in terms of who was the scheme owner, to whom questions could be referred to, and who would take responsibility regarding interpretation and implementation of the guidance. Having a scheme “owner” could enable local commissioners and providers to plan ahead, ask questions and, therefore, eliminate any confusion that might arise given misunderstanding. Providers suggested enhanced communication with all stakeholders (CRGs, regional commissioning hubs, NHS England, clinicians) and clear ownership would help maximise benefits: “The third player in the ownership […] are the hubs because the hubs own the contracts. […] they make the incentives bite. So, they have to own the scheme as well, and that may be where we’re weakest” [C4].

#### Timeliness and time constraints

Successful implementation requires sufficient time for providers to discuss, choose and think through the implementation process. In particular, contracting delays impact not only the amount of time available to affect change, but also the payment incentives. Providers felt particularly challenged during the period when we were undertaking interviews: “This year’s paperwork came through quite late so hit decision of selecting schemes, it was worse than ever. The draft is expected in December, so you’ve got the last quarter until April to have those conversations and you can hit the ground for April 1st, but this year was an absolute fiasco. As a consequence, the first triggers of each scheme had to be amended, probably making it 3 payments instead of 4.” [P4].

Additionally, many participants reported that the scheme aggravated tensions at the provider level, including those with respect to time constraints, which led to either a lack of engagement or increased workload particularly for nurses. “You’ve got to introduce new forms, new processes and you are adding time into an already overly stretched nursing environment” [P5].

#### Flexibility of the schemes

Despite these issues the perception was that there was generally a good relationship between local commissioners and providers. This respect supported a degree of flexibility in the contracting process. The flexibility of the scheme, including the ability to have targets that were specific to providers and their performance were valued by providers (“Sometimes we have to ask commissioners to reword something slightly based on current needs/situation.” [P6]). However, there was concern that providers were experiencing less flexibility and autonomy: "Initially providers were given a pick list from the commissioner, and providers got back and say we’ll do these. Over the years commissioners now say ‘we want you to do this’, so there’s less choice." [P3].

#### Credibility of incentive schemes

If this inflexibility in the contracting process continues, then providers may regard PSS CQUIN schemes as just another commissioning tool (“Innovation and quality improvement make clinicians excited. But sometimes it feels CQUINs are for the NHS the equivalent to interest rates—the only thing Governments can change.”* [P8]*), which might result in the schemes becoming less effective.

Another challenge to a scheme’s credibility and effectiveness is that some Trusts are receiving payments for meeting the targets but not for activities that ultimately benefit patients: “And there’s always the danger with CQUIN schemes that people will comply with it so that they’ll do something that gets the money, but it won’t actually achieve the benefit. …Then there’s the other group of Trusts that have just done it to tick a CQUIN box and get the money.” [C5]. Such behaviour undermines the credibility of any P4P scheme, perhaps suggesting why there is limited evidence of effectiveness for these PSS CQUIN schemes.

#### Sustainability

A key design element of the PSS CQUIN schemes is that they provide an initial incentive to promote innovation or quality improvement that it is intended will become part of standard care at the end of the scheme. This relies both on smooth implementation and a seamless exit strategy. The initial implementation is seen as under-resourced from the commissioners’ perspective: “I think—because it’s such a broad thing to get your head around, I think they’re under-resourced from the point of view of having national oversight of the whole process.” [C5] While providers perceive that they need to constantly look for new funding opportunities: “We are always looking for the new round of funding—time consuming instead of focusing on the change. Also making people redundant is part of this challenge.” [P4].

These views underscore the need for support to ensure sustained involvement of all stakeholders and staff involved in the schemes. This included support beyond the life of the scheme, many participants thought there was a need for proper exit strategies; this appeared to be an overlooked component of the schemes: “It’s the exit strategy that is key for schemes to be self-sustainable, and usually this link is missing” [P8].

## Discussion

### Main findings

Pay-for-Performance (P4P) schemes have become increasingly common internationally, yet evidence of their effectiveness remains ambiguous. The PSS CQUIN P4P programme was introduced to incentivise hospital providers to improve the quality of specialised services in England. We conducted an evaluation of the programme between 2016/2017 and 2018/2019. Our results suggest that the programme had little effect on improving the quality of specialised care, except for reducing delayed hospital discharges from Adult Critical Care. However, the definition of this outcome does leave it susceptible to gaming by providers, and so we cannot entirely rule out the possibility that this effect is driven by changes in reporting rather than reflecting real changes to patient care. The corresponding economic impacts that we estimate represent the upper bound of any likely realised cost savings, as they reflect a scenario under which the estimated impacts on discharge are all real rather than reflecting any gaming of the outcome measure. Depending upon the perspective taken, the incentive payments awarded to providers could either be viewed as costs of the programme or cost savings associated with the schemes (when providers did not achieve 100% compliance). Our qualitative evaluation offers insights as to why the schemes were not effective: design features of the PSS CQUIN programme were considered by key stakeholders as barriers to achieving success.

### Interpretation of findings

In the previous work, we assessed the design of PSS CQUIN against best practice derived from a review of the theoretical and empirical economics literature. In particular, we assessed the scheme design relative to (1) structure vs. process vs. outcome incentives, (2) bonus or penalty, (3) size of payment, (4) unintended consequences, (5) frequency of payments, (6) linear vs. non-linear payments, (7) hospital vs. individual provider payments, (8) absolute vs. relative performance payments, (9) public reporting, (10) mandatory vs. voluntary participation and (11) programme specific data collection. We found that the scheme was largely designed in line with best practice in the literature, although we noted that negotiations with commissioners could be resource intensive for providers.

A further point to consider is whether the incentivised dimensions of quality align well with the utility functions of local decision-makers. In general, each PSS CQUIN scheme was accompanied by a document that explained why a certain measure of performance was incentivised, often with references to evidence related to best practice that could back up the commissioner’s choice of performance indicators. We, therefore, argue that we expect the schemes to be aligned in the provider and the commissioner utility functions as they both depend on the health of patients.

It may, therefore, appear surprising that the scheme did not seem to show much effect on the incentivised activities. Although the incentive payments generally covered more than average costs to providers (or “typical” provider costs in PSS CQUIN documentation), the payments could still be below the marginal costs which is the level required to induce efforts from providers to make improvements in their performance. Average and marginal costs could differ due to time constraints (e.g. availability of clinicians in finding time for service reconfiguration), additional costs of implementing the scheme, and capacity constraints. In addition, the qualitative analysis points to a few potential explanations.

The design of the PSS CQUIN programme is highly complex, and every scheme is unique in its design. For some outcomes under a given scheme, different incentives were applied to different providers. For instance, the payment formulas for the primary outcome under one of the two trauma schemes (TR1) in 2016/2017 differed across four types of providers (where the type is determined by the size of their contract values with NHS England). Such a complex design might well reflect the heterogeneous outcomes that were incentivised, the complex nature of specialised hospital services, and variations of performance between providers. However, accommodating this complexity added considerable burden when implementing the programme. Our qualitative evaluation revealed that some providers found it difficult to understand what they were expected to achieve. Understanding the target has been identified as being key to the success of P4P [15].

The PSS CQUIN schemes also had short timescales for providers to deliver quality improvements. The programme involved frequent payments to providers when different triggers were met. Among the ten schemes in this study, the minimum time period over which performance was measured was three months for five schemes, six months for one scheme, and 12 months for four schemes. While the literature suggests that frequent payments are a preferred payment design [15, 16], the problem with frequent payments under PSS CQUIN is that the programme is dynamic. Even for schemes that lasted for three years, the incentivised outcomes were different in each financial year [11]. As a result, there was little time for providers to catch up if they failed to achieve the incentivised outcomes. The challenges faced by providers and finance/contract staff in NHS Trusts with the PSS CQUIN timelines were commonly expressed in interviews.

Most of the PSS CQUIN schemes incentivised specific process activities [11]. This design is well supported in the literature, which suggests that incentivising process is more effective at inducing effort than linking incentives directly to outcomes unless the outcome is clearly attributable to process activities and clearly understood by providers [17, 18]. The rationale is that improvements in process measures should translate to outcome measures in the end [19, 20]. The justification for the choice of the process measures was often missing in the PSS CQUIN schemes documentation [11]. As such, the link between the selected process measures and intended improvements in outcomes was not always clear. A few schemes also linked payment to the introduction of structures. However, there is limited evidence in the literature on how improvements in structure might be translated to better processes and outcomes [18]. In our qualitative evaluation, some providers raised doubts about whether the programme incentivised activities that result in genuine improvements in healthcare services and ultimately patients’ health.

### Strengths and limitations

Our quantitative evaluation applied a robust design to assess the effectiveness of the PSS CQUIN programme that was introduced in a non-randomised setting. In most of the analyses we used large administrative data sets collected at the patient level, allowing adjustment for changes in the patient composition over time. The analyses applied hospital fixed effects to control for unobserved heterogeneity between providers. With propensity score matching we attempted to address any potential self-selection of providers into specific schemes. A strength of our qualitative evaluation was the systematic approach used in sampling, data collection, and analysis. In addition, purposeful sampling was used to select a wide range of providers with different experiences.

Our study has some limitations. We were unable to quantitively evaluate the impact of all PSS CQUIN schemes in place during our study period because some schemes lacked (comparable) data on outcomes, in particular those related to patients’ quality of life and experience. Another limitation in the quantitative evaluation was the difficulty in identifying the exact patient population targeted due to lack of access to scheme specific data sets. Those evaluations had to rely on approximations of the target populations using HES data. Finally, we cannot rule out the possibility that providers gamed on timely discharges from the Adult Critical Care in TR1 scheme, and that the improvement we find is merely reflecting a change in the timing of the recording of a patient being ready for discharge. In fact, the TR1 background documentation recognised this possibility, although the scheme designers did not find it likely due to the pressure that exists on critical care capacity. For this reason, scheme designers suggested also monitoring night discharges and urgent operations cancelled which were expected to be positively affected by true improvements in timely discharges. We did not, however, find a statistically significant effect of the incentive programme on these outcomes. Our cost estimates likely underestimate the full costs of the programme to commissioners because, for example, no information was available on the scheme implementation costs apart from two schemes, and no information on the time of local NHS England staff based within the regional hubs spent on the PSS CQUIN programme was available. A limitation of our qualitative evaluation was the possibility of recall bias. While we aimed to evaluate schemes between 2016/2017 and 2018/2019, the interviews undertaken in 2018 and 2019 often focused on current experiences rather than past ones, and general rather specific issues. Furthermore, although data saturation was reached within themes, a broader sample and wider representation from diverse providers would have added more plurality of views and additional richness to our qualitative analyses.

## Conclusion

This study provides an evaluation for one of the first nationwide P4P incentive programmes on specialised hospital services. Our evaluation found that the PSS CQUIN schemes had limited impacts on the quality of specialised services in England between 2016/2017 and 2018/2019. These results may be explained by the size of the incentive payments, issues related to the complexity in the design of the schemes, and issues around ownership, contracting and flexibility which made the implementation of these incentive schemes challenging.

### Supplementary Information

Below is the link to the electronic supplementary material.

Supplementary file1 (DOCX 42 KB)

## Data Availability

Most of the data that support the findings of this study are not publicly available. Our use of Hospital Episode Statistics (HES) for the current study was covered by a contract with the NHS Digital. We also used a few centrally collected datasets in this study. The permission for data access was covered by a Confidentiality Agreement with NHS England.
