# Supplementary material for: Pay-for-Performance incentives for specialised services in England: a mixed methods evaluation
Source: Eur J Health Econ. 2023 Oct 13;25(5):857–76. doi: 10.1007/s10198-023-01630-6 (PMC11192700; doi:10.1007/s10198-023-01630-6)
Supplement: Supplementary file 1 — Supplementary file1 (DOCX 42 KB) [file 10198_2023_1630_MOESM1_ESM.docx]

**Supplementary material**

**The European Journal of Health Economics**

**Pay-for-Performance incentives for specialised services in England: a mixed methods evaluation**

Yan Feng ^1*^, Søren Rud Kristensen ^2,3^, Paula Lorgelly ^4,5^, Rachel Meacock ^6^, Alberto Núñez-Elvira ^2^, Marina Rodés-Sánchez ^7^, Luigi Siciliani ^8^, Matt Sutton ^6^

^1^ Centre for Evaluation and Methods, Queen Mary University of London, London, UK

^2^ Institute of Global Health Innovation, Imperial College London, London, UK

^3^ [Danish Centre for Health Economics](https://portal.findresearcher.sdu.dk/en/organisations/dache-dansk-center-for-sundheds%C3%B8konomi), University of Southern Denmark, Odense, Denmark

^4^ Faculty of Medical and Health Sciences and School of Business, University of Auckland, Auckland, New Zealand

^5^ Department of Applied Health Research, University College London, London, UK

^6^ Health Organisation, Policy and Economics, University of Manchester, Manchester, UK

^7^ Office of Health Economics, London, UK

^8^ Department of Economics and Related Studies, University of York, York, UK

***Corresponding author**: [yan.feng@qmul.ac.uk](mailto:yan.feng@qmul.ac.uk)

**Supplementary Appendix 1: Qualitative Analysis – Data collection schedules**

1. Interview Schedule [Department of Health and Social Care and NHS England staff]

Question topics to be refined and expanded based on results of literature review, and to be adapted according to information from ongoing interviews and interviewee role.

Logistics around agreement/consent and recording. Introduction to Office of Health Economics (OHE) and scope of the study. Ascertain background of interviewee (speciality, experience), current role and relationship with PSS CQUIN schemes.

**Topics**

- History of PSS CQUIN schemes design and implementation
  - What informed which kind of financial incentives to implement
  - How were specialised areas selected/deselected
    - Achievement in change of behaviour
  - How were performance measures for each scheme selected
  - How were thresholds and payment mechanisms selected
  - Roles of accountability: from DH to NHS England
  - The future of PSS CQUIN schemes
- Costs of delivering PSS CQUIN
  - What’s involved in implementing the whole PSS CQUIN
    - Jobs and opportunity costs of staff
    - External/internal contracts
    - IT systems
    - Were other ways of changing behaviour considered
- Perceptions regarding the implementation of the schemes
  - Decision on what schemes to implement: from CCG to Trusts
  - How well do NHS Commissioners and Trusts implement schemes
- Perception of uptake and satisfaction
  - What was the expectation on uptake, has it been met
  - Perception on time horizon considered
  - As scheme designers are they happy, why/why not
  - Do they think Commissioners and Trusts are happy, why/why not
    - Evaluation of satisfaction
- Challenges or barriers to implementation and ways of improving
- Thoughts on the sustainability of PSS CQUIN schemes
  - Could the scheme be broadened
  - Wider use of financial incentives more broadly
- Anything important that may be missing / you would like to add.

2. Interview Schedule [Local NHS Commissioners]

Question topics to be refined and expanded based on results of literature review, and to be adapted according to information from ongoing interviews and interviewee role.

Logistics around agreement/consent and recording. Introduction to OHE and scope of the study.

Ascertain background of interviewee (speciality, experience), current role and relationship with PSS CQUIN schemes.

**Topics**

- What was your level of involvement in designing PSS CQUIN schemes
  - Do you have flexibility or choice in their implementation
- Perceptions on implementation
  - Have you had feedback from staff, including clinicians and patients on success or effect of the scheme (do you have formal feedback you could share?)
  - Have the schemes been accepted by staff, do they think they are worthwhile implementing
  - How long has implementation taken
- What performance monitoring mechanisms are in place
- How has contracting compared with other schemes or systems
- Perception of evolution of treatment patterns or quality of care
- Perception of uptake and satisfaction
  - What was the expectation on the scheme, has it met your expectations
  - Do they think Trusts are happy, why/why not
- Challenges or barriers to implementation and ways of improving the scheme
- Thoughts on sustainability of PSS CQUIN schemes
  - Could the scheme be broadened, what impact what that have on you
  - wider use of financial incentives more broadly
- Differences between what is funded vs. what is achievable
- Specific financial stability challenges
- Benefits/disadvantages of PSS CQUIN schemes for staff

3. Interview Schedule [National Programmes of Care]

Logistics around agreement/consent and recording.

Introduction to OHE and scope of the study.

Ascertain background of interviewee (speciality, experience), current role and relationship with PSS CQUIN schemes.

**Topics**

- Reflect on the make-up of the PoC: o what is the role of the different participants?
  - What else do you do aside from designing CQUIN schemes?
  - Do different members of the group engage differently in the various tasks?
- What is your level of involvement in designing PSS CQUIN schemes?
- Phases in the design process (from the moment an idea gets to you until a new scheme is sent to NHS England).
- Challenges/barriers when thinking about certain aspects of the schemes: to linking theory to practice,
  - deciding payment methods,
  - triggers,
  - where flexibility will be allowed
  - Trusts’ heterogeneity.
- Reflections on schemes ownership
- Once a scheme has been approved, o do you have contact with commissioners and providers?
  - do you hear back from NHS England regarding commissioners/providers experiences when implementing the schemes?
  - do you seek feedback after implementation for editing and updating the schemes in advance of the next contracting round?
- What monitoring mechanisms are in place (if any)?
- Benefits/disadvantages PSS CQUIN schemes for staff and patients
- Reflections on sustainability: o embedding new behaviours in normal practice,
  - exit strategies,
  - financial stability challenges.

4. Interview Schedule [Clinical Reference Groups]

Logistics around agreement/consent and recording.

Introduction to OHE and scope of the study.

Ascertain background of interviewee (speciality, experience), current role and relationship with PSS CQUIN schemes.

Please focus on your role as CRG member when answering the questions below, unless specified by the interviewer.

**Topics**

- Reflect on the make-up of the CRG:
  - what is the role of patients?
  - What else do you do aside from designing CQUIN schemes?
  - Do different members of the group engage differently in the various tasks?
- What is your level of involvement in designing PSS CQUIN schemes?
- Phases in the design process (from the moment an idea gets to you until a new scheme is sent to NHS England).
- Challenges/barriers when thinking about certain aspects of the schemes:
  - linking theory to practice,
  - deciding payment methods,
  - triggers,
  - where flexibility will be allowed
  - Trusts’ heterogeneity.
- Reflections on schemes ownership
- Once a scheme has been approved,
  - do you have contact with commissioners and providers?
  - do you hear back from NHS England regarding commissioners/providers experiences when implementing the schemes?
  - do you seek feedback after implementation for editing and updating the schemes in advance of the next contracting round?
- What monitoring mechanisms are in place (if any)?
- Benefits/disadvantages PSS CQUIN schemes for staff and patients
- Reflections on sustainability:
  - embedding new behaviours in normal practice,
  - exit strategies,
  - financial stability challenges.

5. Focus Group Schedule [Providers]

**Welcome**

“Many thanks for coming along this morning/afternoon.”

*[Ascertain background of interviewee (speciality, experience), and current role]*: *“*To formally introduce myself – I’m XXX and I work for XXXX.”

*[Introduction to organiser and scope of the study]:* “This project is a result of a collaboration between Imperial College London, The Office of Health Economics, The University of Manchester, and the University of York. The objective is to gain insight on the challenges and barriers arising with the scheme’s implementation, and get an overview of the factors influencing uptake and sustainability.”

*[Logistics around agreement/consent and recording]*: “The discussion is being tape-recorded. This is standard market research procedure and is to ensure accuracy – so I do not have to try to remember what you have said – and for analysis purposes only. The recordings will not be passed to any third party not associated with the research project, and I assure you that none of your comments will be attributed to you by name.”

“The research is being conducted in accordance with the Code of Conduct of the Market Research Society (MRS) and also with the Data Protection Act. This means that everything you say here today is confidential and will not be attributed to you personally.”

“I want to emphasise that we are looking for your views. There are no right or wrong answers. You may not agree with each other and that is fine. The whole purpose of getting a group of people in a room together is to get an exchange of views. I hope you will all contribute to the discussion. The discussion will last around 120 minutes.”

“OK – before we start let’s do introductions…”

**Warm-Up/Reintroductions**

Introductions

- Name
- Current role
- Experience with PSS CQUIN schemes
- Level of involvement in designing PSS CQUIN schemes: did you have flexibility or choice in their implementation

**Group discussion about perceptions on implementation**

“To begin with, we would like to learn your views about the implementation process. How did you live the process? And the rest of staff?”

[Questions to be answered by all participants as part of a group discussion.]

Areas to be approached:

- Feedback from staff, including clinicians and patients on success or effect of the scheme
- Whether implementation is worthwhile
- Duration
- Benefits/disadvantages of PSS CQUIN schemes for staff
- How has contracting compared with other schemes or systems
- Perception of evolution of treatment patterns or quality of care

**Group discussion about challenges and barriers**

“We would like your views about challenges and barriers to implementation, as well as ways of improving the scheme.”

**Group discussion about uptake and satisfaction**

“We would like to know what your expectation on the scheme was, and whether it has met your expectations. Do you think Trusts are happy? Why?”

**Group discussion about sustainability of PSS CQUIN schemes**

“Before we finish, we would like to know about your perception on the sustainability of PSS CQUIN schemes.”

[Questions to be answered by all participants as part of a group discussion.]

Areas to be approached:

- Could the scheme be broadened, what impact what that have on you
- wider use of financial incentives more broadly
- Differences between what is funded vs. what is achievable
- Specific financial stability challenges

**Debrief**

Ask for feedback and assessments of how difficult the various tasks were

Whether any of the questions made the participants feel upset or uncomfortable

**Wrap-up and close**

Return to main questions to check if participants want to add further comments.

Thank participants and close.

**Supplementary Appendix 2(a): Descriptive statistics of treatment and control providers for schemes GE1, GE2, CA1, and CA3.**

|  | GE1 |  | GE2 |  | CA1 |  |  | CA3 |  |  |
| --- | --- | --- | --- | --- | --- | --- | --- | --- | --- | --- |
|  | Treatment group (all) | Control group (all) | Treatment group (all) | Control group (all) | Treatment group (all) | Control group (all) | Control group (matched) | Treatment group (all) | Control group (all) | Control group (matched) |
|  | Mean  (SE) | Mean  (SE) | Mean  (SE) | Mean  (SE) | Mean  (SE) | Mean  (SE) | Mean  (SE) | Mean  (SE) | Mean  (SE) | Mean  (SE) |
| N of beds | 942.726 | 785.588 | 693.952 | 705.718 | 932.108 | 720.545 | 869.762 | 894.044 | 712.893 | 808.749 |
|  | (70.803) | (99.962) | (51.577) | (36.790) | (111.013) | (28.166) | (73.958) | (61.578) | (32.189) | (58.120) |
| Prop of doctors | 12.246 | 12.274 | 11.030 | 11.011 | 12.285 | 11.249 | 12.238 | 11.921 | 11.126 | 11.930 |
|  | (0.394) | (0.521) | (0.471) | (0.261) | (0.488) | (0.199) | (0.629) | (0.379) | (0.228) | (0.399) |
| MFF tariff | 1.096 | 1.088 | 1.083 | 1.081 | 1.096 | 1.076 | 1.109 | 1.095 | 1.074 | 1.094 |
|  | (0.013) | (0.018) | (0.011) | (0.007) | (0.019) | (0.006) | (0.019) | (0.013) | (0.006) | (0.012) |
| Foundation Trust | 0.674 | 0.667 | 0.686 | 0.641 | 0.476 | 0.672 | 0.476 | 0.656 | 0.650 | 0.656 |
|  | (0.072) | (0.126) | (0.080) | (0.045) | (0.112) | (0.043) | (0.112) | (0.085) | (0.047) | (0.085) |
| Teaching hospital | 0.047 | 0.000 | 0.171 | 0.205 |  |  |  | 0.219 | 0.223 | 0.281 |
|  | (0.032) | (0.000) | (0.065) | (0.037) |  |  |  | (0.074) | (0.041) | (0.081) |
| London | 0.233 | 0.133 | 0.114 | 0.171 | 0.238 | 0.131 | 0.381 | 0.156 | 0.136 | 0.125 |
|  | (0.065) | (0.091) | (0.055) | (0.035) | (0.095) | (0.031) | (0.109) | (0.065) | (0.034) | (0.059) |
| N of obs | 43 | 15 | 35 | 117 | 21 | 120 | 21 | 32 | 103 | 32 |
| PSM applied | No |  | No |  | Yes |  |  | Yes |  |  |

^a^ MFF = Market Forces Factor. PSM = Propensity Score Matching.

^b^ Descriptive statistics for schemes MH2 and MH4 are not included due to data availability.

**Supplementary Appendix 2(b): Descriptive statistics of treatment and control providers for schemes IM1, TR1, TR3 and WC5.**

|  | IM1 |  |  | TR1 |  |  |  | TR3 |  |  | WC5 |  |  |
| --- | --- | --- | --- | --- | --- | --- | --- | --- | --- | --- | --- | --- | --- |
|  | Treatment group (all) | Control group (all) | Control group (matched) | Treatment group (all) | Treatment group (matched) | Control group (all) | Control group (matched) | Treatment group (all) | Control group (all) | Control group (matched) | Treatment group (all) | Control group (all) | Control group (matched) |
|  | Mean  (SE) | Mean  (SE) | Mean  (SE) | Mean  (SE) | Mean  (SE) | Mean  (SE) | Mean  (SE) | Mean  (SE) | Mean  (SE) | Mean  (SE) | Mean  (SE) | Mean  (SE) | Mean  (SE) |
| N of beds | 1063.110 | 1131.174 | 1098.093 | 829.785 | 831.454 | 637.265 | 664.936 | 1059.700 | 838.222 | 1130.325 | 1103.451 | 972.815 | 1011.721 |
|  | (161.438) | (79.811) | (104.833) | (50.445) | (51.153) | (41.347) | (42.569) | (134.034) | (95.795) | (104.330) | (92.238) | (80.922) | (89.776) |
| Prop of doctors | 11.219 | 13.751 | 13.025 | 11.827 | 11.792 | 10.190 | 10.940 | 11.933 | 12.465 | 12.670 | 12.101 | 12.473 | 12.297 |
|  | (0.664) | (0.421) | (0.235) | (0.289) | (0.291) | (0.393) | (0.315) | (0.437) | (0.434) | (0.619) | (0.634) | (0.443) | (0.498) |
| MFF tariff | 1.062 | 1.114 | 1.098 | 1.080 | 1.081 | 1.076 | 1.079 | 1.060 | 1.090 | 1.063 | 1.100 | 1.071 | 1.070 |
|  | (0.016) | (0.023) | (0.027) | (0.009) | (0.009) | (0.008) | (0.008) | (0.023) | (0.015) | (0.012) | (0.023) | (0.012) | (0.013) |
| Foundation Trust | 0.583 | 0.438 | 0.500 | 0.635 | 0.639 | 0.739 | 0.770 | 0.727 | 0.720 | 0.636 | 0.632 | 0.682 | 0.684 |
|  | (0.149) | (0.128) | (0.151) | (0.061) | (0.062) | (0.053) | (0.054) | (0.141) | (0.092) | (0.152) | (0.114) | (0.102) | (0.110) |
| Teaching hospital | 0.417 | 0.750 | 0.667 | 0.159 | 0.164 | 0.246 | 0.279 | 0.091 | 0.080 | 0.182 | 0.053 | 0.182 | 0.105 |
|  | (0.149) | (0.112) | (0.142) | (0.046) | (0.048) | (0.052) | (0.058) | (0.091) | (0.055) | (0.122) | (0.053) | (0.084) | (0.072) |
| London | 0.083 | 0.312 | 0.250 | 0.190 | 0.180 | 0.130 | 0.148 | 0.091 | 0.200 | 0.091 | 0.316 | 0.091 | 0.105 |
|  | (0.083) | (0.120) | (0.131) | (0.050) | (0.050) | (0.041) | (0.046) | (0.091) | (0.082) | (0.091) | (0.110) | (0.063) | (0.072) |
| N of obs | 12 | 16 | 12 | 62-63 | 61 | 66-69 | 61 | 11 | 25 | 11 | 19 | 22 | 19 |
| PSM applied | Yes |  |  | Yes |  |  |  | Yes |  |  | Yes |  |  |

^a^ MFF = Market Forces Factor. PSM = Propensity Score Matching.

^b^ Descriptive statistics for schemes MH2 and MH4 are not included due to data availability.

^c^ The number of observations in TR1 scheme: treatment group (all) for prop of doctors and MFF tariff (N=62), treatment group (all) for N of beds, Foundation Trust, Teaching hospital and London (N=63), control group (all) for N of beds (N=66), control group (all) for prop of doctors and MFF tariff (N=68), control group (all) for Foundation Trust, Teaching hospital and London (N=69).

**Supplementary Appendix 3: Mean values for each outcome before and after the implementation of PSS CQUIN by treatment and control groups**

|  | Treatment group | | Control group | |
| --- | --- | --- | --- | --- |
|  | Before | After | Before | After |
| GE1: Clinical Utilisation Review | | | | |
| LOS Emergency admissions | 8.675 | 8.657 | 8.515 | 8.253 |
| Log of LOS Emergency admissions | 1.652 | 1.657 | 1.662 | 1.646 |
| LOS elective admissions | 5.550 | 5.534 | 5.238 | 5.176 |
| Log of LOS elective admissions | 1.323 | 1.322 | 1.295 | 1.283 |
| Number of emergency admissions | 1,328.511 | 1,635.634 | 1,799.032 | 2,273.251 |
| Log of number of emergency admissions | 6.269 | 6.892 | 6.416 | 7.042 |
| Number of unique patients treated | 1,042.326 | 1,245.773 | 1,286.407 | 1,536.692 |
| Log of number of unique patients treated | 6.304 | 6.924 | 6.429 | 6.966 |
| GE2: Activation System for Patients with Long Term Conditions | | | | |
| Number of HIV admissions | 11.651 | 14.044 | 10.690 | 12.455 |
| Log of number of HIV admissions | 1.968 | 2.129 | 1.678 | 1.832 |
| Number of respiratory disease emergency admissions | 691.939 | 996.449 | 688.838 | 923.926 |
| Log of number of respiratory disease emergency admissions | 6.069 | 6.487 | 5.992 | 6.265 |
| Number of renal disease emergency admissions | 139.993 | 229.238 | 182.617 | 181.345 |
| Log of number of renal disease emergency admissions | 4.286 | 4.841 | 4.667 | 4.956 |
| Emergency readmissions within 30 days (respiratory patients) | 0.108 | 0.102 | 0.105 | 0.106 |
| CA1: Enhanced Supportive Care for Advanced Cancer Patients | | | | |
| Number of chemotherapy/radiotherapy treatments | 1.508 | 1.494 | 1.474 | 1.479 |
| Number of emergency admissions | 0.255 | 0.231 | 0.236 | 0.199 |
| LOS Emergency admissions | 3.738 | 3.193 | 3.531 | 3.038 |
| CA3: Optimising Palliative Chemotherapy Decision Making | | | | |
| Deaths within 30 days of last chemotherapy | 0.862 | 0.870 | 0.850 | 0.859 |
| IM1: Reducing Cardiac Surgery Non-Elective Inpatient Waiting | | | | |
| Days within 7 (Pooled) | 0.384 | 0.511 | 0.475 | 0.535 |
| LOS (CABG) | 19.766 | 17.583 | 19.960 | 18.873 |
| Mortality within 30 days | 0.247 | 0.253 | 0.030 | 0.030 |
| Any patient safety incidents | 0.131 | 0.154 | 0.130 | 0.162 |
| TR1: Adult Critical Care Timely Discharge | | | | |
| Delayed discharges <4 hours | 0.521 | 0.544 | 0.529 | 0.498 |
| Night discharges | 0.172 | 0.185 | 0.201 | 0.210 |
| Total urgent operations cancelled | 1.190 | 2.129 | 2.159 | 1.724 |
| Log of total urgent operations cancelled | 1.182 | 1.586 | 1.499 | 1.357 |
| TR3: Spinal Surgery Networks | | | | |
| Number of spinal surgeries | 8.898 | 8.302 | 7.454 | 6.889 |
| WC5: Neonatal Community Outreach | | | | |
| LOS of newborns | 5.999 | 6.176 | 6.716 | 6.784 |
| MH2: Recovery Colleges for patients who receive low and medium secure mental health services | | | | |
| LOS | 555.785 | 179.422 | 555.979 | 186.564 |
| Log of LOS | 5.882 | 4.736 | 5.863 | 4.758 |
| MH4: Discharge and Resettlement planning programme in mental health (to remove hold-ups in discharge) | | | | |
| LOS children and adolescents | 87.845 | 84.747 | 85.089 | 76.236 |
| Log of LOS children and adolescents | 3.977 | 3.981 | 4.052 | 3.857 |
| LOS adults | 175.748 | 191.716 | 186.531 | 185.531 |
| Log of LOS adults | 4.879 | 5.000 | 5.030 | 5.004 |

^a^ LOS = length of stay. CABG = Coronary Artery Bypass Graft
